# Distribution of COVID-19 Morbidity Rate in Association with Social and Economic Factors in Wuhan, China: Implications for Urban Development

**DOI:** 10.3390/ijerph17103417

**Published:** 2020-05-14

**Authors:** Heyuan You, Xin Wu, Xuxu Guo

**Affiliations:** 1 School of Public Administration, Zhejiang University of Finance and Economics, Hangzhou 310018, China; wx1012@zufe.edu.cn (X.W.); xuxug42@zufe.edu.cn (X.G.); 2 Department of City and Regional Planning, University of North Carolina at Chapel Hill, Chapel Hill, NC 27514, USA

**Keywords:** social and economic factors, spatial regression analysis, COVID-19, morbidity rate, Wuhan city

## Abstract

Social and economic factors relate to the prevention and control of infectious diseases. The purpose of this paper was to assess the distribution of COVID-19 morbidity rate in association with social and economic factors and discuss the implications for urban development that help to control infectious diseases. This study was a cross-sectional study. In this study, social and economic factors were classified into three dimensions: built environment, economic activities, and public service status. The method applied in this study was the spatial regression analysis. In the 13 districts in Wuhan, the spatial regression analysis was applied. The results showed that: 1) increasing population density, construction land area proportion, value-added of tertiary industry per unit of land area, total retail sales of consumer goods per unit of land area, public green space density, aged population density were associated with an increased COVID-19 morbidity rate due to the positive characteristics of estimated coefficients of these variables. 2) increasing average building scale, GDP per unit of land area, and hospital density were associated with a decreased COVID-19 morbidity rate due to the negative characteristics of estimated coefficients of these variables. It was concluded that it is possible to control infectious diseases, such as COVID-19, by adjusting social and economic factors. We should guide urban development to improve human health.

## 1. Introduction

The emergence of COVID-19 in Wuhan was reported at the end of 2019. The previously unknown coronavirus SARS-CoV-2 has been identified as the cause. The outbreak of COVID-19 has become a global health concern [1]. Understanding the COVID-19 would help control the spread of these infectious diseases. But there are too many questions about the outbreak of COVID-19 since it is a new infectious disease. Infectious diseases are ‘eco-bio-social’ events influenced by ecological, biological, and social factors [2]. A lot of social and economic factors that change human behavior present challenges for the prevention and control of infectious diseases [3]. These social and economic factors consist of population density, urban/rural setting, urbanization, population growth, land-use change [4,5,6]. Similar to the known infectious diseases, the prevention and control of infectious diseases of COVID-19 may relate to these social and economic factors. Therefore, we should identify these influencing factors and assess the relationships between social and economic factors and the features of the COVID-19 pandemic.

According to the previous researches, social and economic factors have the potential to impact infectious disease dynamics [7]. Ponnambalam et al. (2012) found that H1N1 deaths concentrated in counties with low population density and the high percent of the population aged ≥65 years [8]. Fang et al. (2009) revealed that medical staff density and population density had significant positive influences on the presence of SARS in mainland China [9]. Lowcock et al. (2012) found that school education and living conditions were associated with hospitalization in pandemic H1N1 2009 influenza [10]. Huang et al. (2014) discovered that the tertiary industry and middle school distribution had important impacts on the incidence of hand-foot-mouth disease in China [11]. Mamelund (2011) argued that geographic isolation and urban area relate to the mortality in the 1918–20 influenza pandemic [12]. Besides, COVID-19 can be transmitted through respiratory droplets and contact routes [13,14]. COVID-19 seems to be spreading easily and sustainably in the community. Based on the literature review, we believed that social and economic factors also have impacts on the COVID-19 spread. Because the COVID-19 morbidity rate is a crucial indicator to describe the spread of COVID-19, this study tried to reveal the correlation between social and economic factors and the distribution of the COVID-19 morbidity rate.

Understanding the distribution of COVID-19 morbidity rate in association with social and economic factors can benefit the control of infectious diseases’ transmission in the outbreak of infectious diseases, such as COVID-19. It also helps design a healthy city. The outbreak of COVID-19 started in Wuhan, China. Globally, Wuhan was the most affected city with the COVID-19 pandemic until March 2020. Therefore, Wuhan was selected as the study area in this study. Under this circumstance, this article aimed to analyze the distribution of the COVID-19 morbidity rate in association with social and economic factors in Wuhan, China. We specifically attempted to (1) select the social and economic factors related with COVID-19 spread, (2) quantify the relationships between social and economic factors and COVID-19 morbidity rate, (3) discuss the possible impacts of social and economic factors on COVID-19 morbidity rate, and (4) reveal the implications for urban development.

## 2. Materials and Methods

### 2.1. Study Design

**Figure 1:**
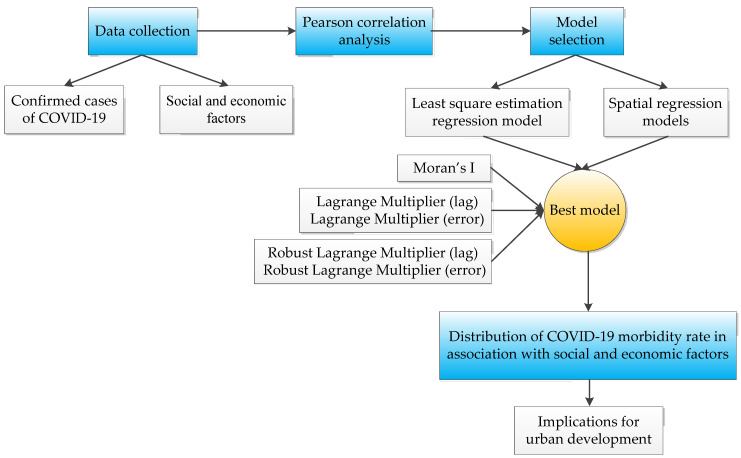
Study design.

This study was a cross-sectional study of the distribution of the COVID-19 morbidity rate in association with social and economic factors. A diagram showing the key study design elements is shown in Figure 1. The spatial regression analysis was applied in the administrative divisions that refer to 13 districts in Wuhan. We collected the data on social and economic factors from statistical yearbooks and the department of land resources. Meanwhile, the number of confirmed cases of COVID-19 in Wuhan was collected in the WeChat official account of the Wuhan government. The bivariate analysis for the relationships of each of these influencing factors and the COVID-19 morbidity rate using Pearson correlation analysis was done. Then, the suitable estimation model was selected from the least square estimation regression model and spatial regression models. Lastly, the implications for urban development were discussed. More details are presented in the Supplemental Material.

### 2.2. Study Area

**Figure 2:**
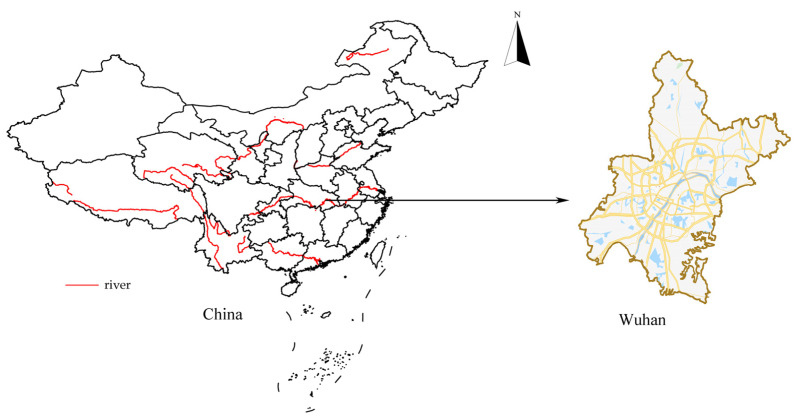
Location of Wuhan, China.

Wuhan city, located in the confluence of the Han River flowing into the Yangtze River, is the capital and most populous city of Hubei Province in central China (Figure 2). Wuhan has humid subtropical with cold winters and wet, hot summers. Wuhan is one of the most important transportation hubs in China. Wuhan railway hub is considered one of the four key railway hubs of China. Wuhan cuisines have special dishes and local snacks of China due to the convenient transportation and long history of Wuhan. In order to make delicacies, various ingredients that are collected from land, rivers, lakes, and sea are provided in the markets in Wuhan. Wuhan’s Huanan seafood market, where wild animals are traded, is considered as the origin of COVID-19 in Wuhan [15]. On 22 February 2020, a total of 46,201 confirmed cases of COVID-19 were reported in Wuhan. 

Administrative divisions of Wuhan currently comprise central districts and suburban-rural districts. Central districts are the populous areas with the developed economy in Wuhan. Central districts include 7 districts: Jiang ‘a District, Jianghan District, Qiaokou District, Hanyang District, Wuchang District, Qingshan District, Hongshan District. Suburban-rural districts include 6 districts: Dongxihu District, Wuhan development zone, including Hannan District, Caidian District, Jiangxia District, Huangpi District, Xinzhou District. In this study, the spatial regression analysis was applied in the administrative divisions that refer to 13 districts in Wuhan.

### 2.3. Data Collection

The morbidity rate has been defined as the proportion of an initially disease-free population that suffers a disease during a specified time interval. Therefore, the COVID-19 morbidity rate is the proportion with which COVID-19 appears in a population. The COVID-19 morbidity rate was defined as the ratio of confirmed cases of COVID-19 to the average population in this study. The number of confirmed cases of COVID-19 in Wuhan was collected in the WeChat official account of the Wuhan government. The deadline date of confirmed cases of COVID-19 was 22 February 2020. At the meeting to advance the work on coordinating the prevention and control of the COVID-19 and economic and social development on 23 February 2020, Chinese President Xi Jinping said that the positive situation of epidemic prevention and control is expanding. Meanwhile, after 22 February 2020, people have been forbidden to leave the house in Wuhan. Therefore, the distribution of confirmed cases of COVID-19 on 22 February 2020, could reveal the spread features in Wuhan. The average population is the arithmetic mean value of the resident population and household registration population since many immigrants left Wuhan before the Spring Festival holiday. In 2020, the original Spring Festival holiday lasted for 7 days from January 24 to January 30, and it has been extended since the COVID-19 pandemic. COVID-19 morbidity rate in Wuhan was 47.56‱ on 22 February 2020, and COVID-19 morbidity rates in different districts are shown in Table 1.

The social and economic factors were classified into three dimensions: built environment, economic activities, and public service status (Table 2). The built environment refers to the human-made environment that provides space for human activity. Population density and construction land distribution have a relationship with the transmission of infectious diseases [8,11]. Meanwhile, community spread exists in the COVID-19 spread [16]. The built environment was classified into three representative parts: population density, construction land area proportion, and average building scale. Economic activities are related to human behavior. It reveals the population aggregation and population flow [17,18,19]. Therefore, economic activities had impacts on the COVID-19 morbidity rate, which could be transmitted through respiratory droplets and contact routes. Economic activities covered GDP per unit of land area, value-added of tertiary industry per unit of land area, and total retail sales of consumer goods per unit of land area. Public service status was selected to describe the public service facility distribution [20,21]. Public service facilities play a crucial role in public health problems caused by infectious diseases [22]. Therefore, public service status was classified into three representative parts: public green space density, hospital density, and aged population density. Data sources for the variables of social and economic factors included the Wuhan Statistical Yearbook (2018), Wuhan Construction Yearbook (2017, 2018), and Wuhan Health Yearbook (2018). Besides, the data of construction land areas were collected from the department of land resources of Hubei province.

### 2.4. Spatial Regression Analysis

In order to avoid estimation deviation caused by spatial dependence, the suitable estimation model was selected from the least square estimation regression model (OLS) and spatial regression models. Classical spatial econometric models included the spatial lag model (SLM) and the spatial error model (SEM). The mathematical expressions of SLM (Equation (1)) and SEM (Equation (2)) were as follows:

(1) $$y=\beta x+\lambda W_{y}+u$$

(2) $$y=\beta x+u,\;u=\rho W_{u}+v$$

where *y* is the COVID-19 morbidity rate, *x* is the variables of social and economic factors, *β* is the coefficient of the independent variable, *W* is the spatial weight, λ is a spatial lag coefficient, *ρ* is the spatial error coefficient, *u* is a residual error, *v* is white noise.

Maximum likelihood estimation was applied in SLM and SEM [23]. The spatial correlations were divided by administrative divisions in Wuhan. The spatial weights matrix quantified the spatial relationships that existed among the features in the dataset. The important transmission routes of COVID-19 include droplets and fomites. It has the transmission ability. Therefore, the spatial weights matrix that could reveal more neighbor relations was selected. Queen contiguity weight was selected as the spatial weights matrix. Common sides of the polygons or common vertices are considered to define the neighbor relation in the queen contiguity weight matrix. The Yangtze River and the Han River cause spatial partitions in Wuhan. There are 11 Yangtze River bridges and 5 tunnels under the Yangtze River. Whereas, there are 11 Han River bridges and 2 tunnels under the Han River. The Yangtze River and the Han River do not block the communication between different regions in Wuhan. Therefore, the Yangtze River and the Han River did not break the neighbor relations among administrative divisions in assessing the spatial weights matrix in the study. The selection between the least square estimation regression model and spatial regression models was based on the significance of Moran’s I. The selection of SLM and SEM was based on the selection criteria proposed by Anselin (2005) [24]. Geoda was selected to analyze spatial regression in this study. The spatial correlation test for the COVID-19 morbidity rate was also performed in Geoda.

## 3. Results

**Figure 3:**
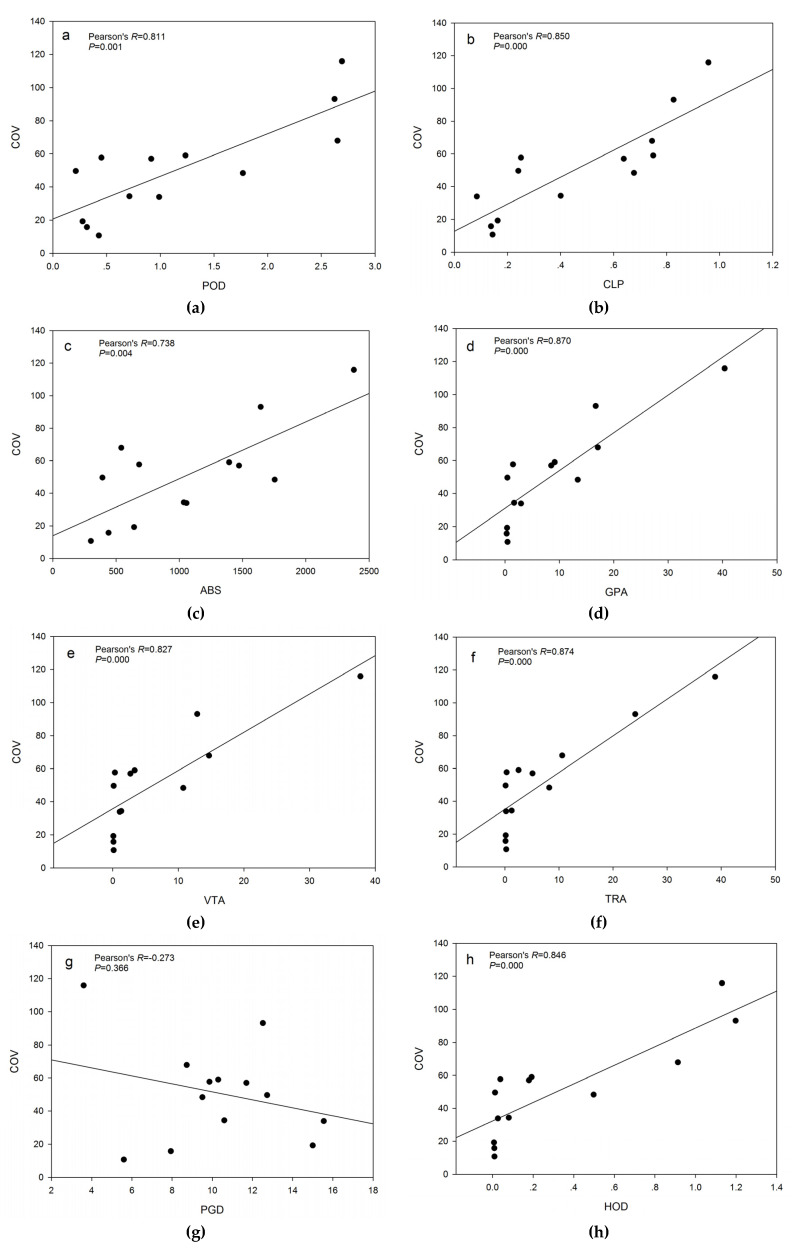

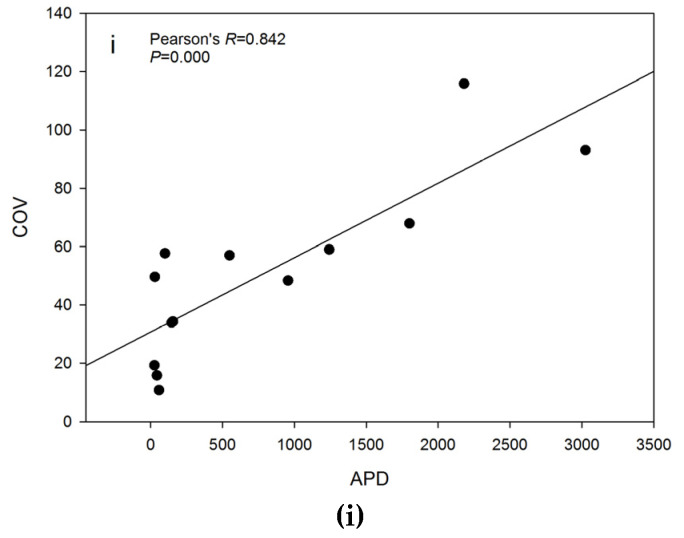
Pearson correlation analysis for the influencing factors and COVID-19 morbidity rate. (**a**) POD (Population density), (**b**) CLP (Construction land area proportion), (**c**) ABS (Average building scale), (**d**) GPA (GDP per unit of land area), (**e**) VTA (Value-added of tertiary industry per unit of land area), (**f**) TRA (Total retail sales of consumer goods per unit of land area), (**g**) PGD (Public green space density), (**h**) HOD (Hospital density), (**i**) APD (Aged population density).

The descriptive statistics of variables are shown in Table 3. The bivariate analysis for the relationships of each of these influencing factors and the COVID-19 morbidity rate using Pearson correlation analysis revealed the strength of the correlation hidden in the results (Figure 3). COVID-19 morbidity rate was significantly related to social and economic factors, except for PGD.

**Figure 4:**
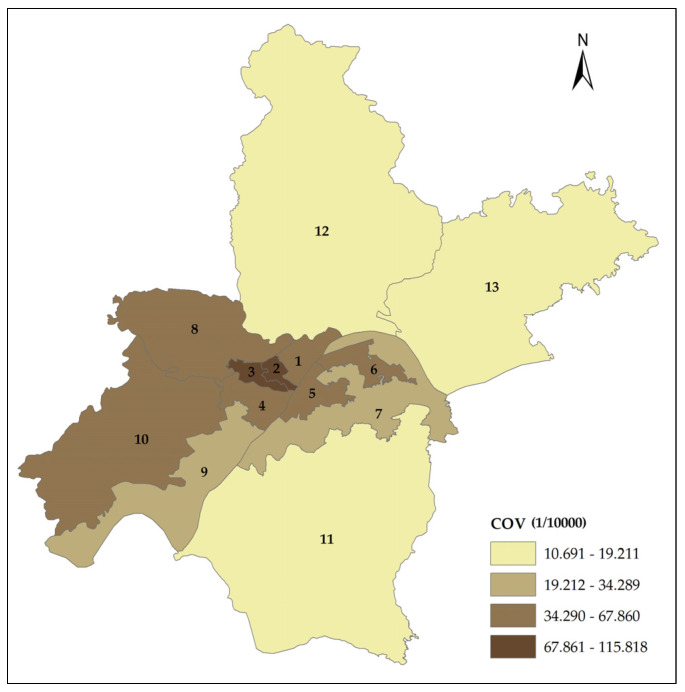
COV (COVID-19 morbidity rate). Spatial distribution of COVID-19 morbidity rate in Wuhan. Administrative divisions: 1 (Jiang’an District), 2 (Jianghan District), 3 (Qiaokou District), 4 (Hanyang District), 5 (Wuchang District), 6 (Qingshan District), 7 (Hongshan District), 8 (Dongxihu District), 9 (Wuhan development zone including Hannan District), 10 (Caidian District), 11 (Jiangxia District), 12 (Huangpi District), 13 (Xinzhou District).

The spatial distribution of the COVID-19 morbidity rate is shown in Figure 4. The result showed that Moran’s I value of COVID-19 morbidity rate was 0.450. In order to assess the *p*-value, a randomization process was selected. A total of 999 permutations were used to construct the reference distribution in the randomization process. The result showed that the *p*-value of the COVID-19 morbidity rate was 0.001. The test supported that there was spatial autocorrelation in the COVID-19 morbidity rate in Wuhan. Therefore, the spatial regression model should be chosen to avoid the estimation bias that was caused by OLS estimation. According to the selection criteria of the SLM model and SEM model that was proposed by Anselin (2005) [24], the SLM model was selected in this study since Lagrange Multiplier (lag) was significant and Lagrange Multiplier (error) was not significant (Table 4). 

Table 4 shows the relationship between the built environment and COVID-19 morbidity rate in Wuhan, China. A significant positive relationship was identified between the POD and the COV. It indicated that high population density could increase the COVID-19 morbidity rate. CLP had a significant positive impact on COV. It implied that the high construction land area proportion could result in the high COVID-19 morbidity rate. Meanwhile, ABS presented a negative relationship with the COV. It suggested that increasing the average building scale could reduce the COVID-19 morbidity rate. Table 4 also presents the relationships between economic activities and the COVID-19 morbidity rate. A significant negative relationship was identified between the GPA and the COV. VTA and TRA had a significant positive impact on the COV. It denoted that economic activities in the land had multiple effects on the COVID-19 morbidity rate. Table 4 shows the relationship between public service status and the COVID-19 morbidity rate. PGD and APD were positive contributors to COV, while HOD was a negative contributor to COV. It implied that high public green space density and aged population density could increase the COVID-19 morbidity rate. High hospital density could reduce the COVID-19 morbidity rate. In conclusion, social and economic factors had an important impact on the distribution of the COVID-19 morbidity rate in Wuhan, China.

## 4. Discussion

### 4.1. Possible Impacts of Social and Economic Factors on COVID-19 Morbidity Rate

Several causes may explain the observed relationships between social and economic factors on the COVID-19 morbidity rate. Specifically, areas with high population density imply more people live in per unit of area. It increases the contact among the people. The important transmission routes of COVID-19 include droplets and fomites during close unprotected contact between an infector and infectee [13]. Therefore, the high population density will accelerate the diffusion of COVID-19 in the crowd. These results are in line with the previous research of Chhikara et al. (2020), who discussed that increased population density increased the possibility of transmission of COVID-19 [25]. Meanwhile, the urban-rural segmentation is conducive to reducing the transmission of infectious diseases between urban and rural areas. An urban-rural effect was also observed in the 1918 influenza pandemic [26]. High construction land area proportion results in the fragmentation of rural areas. The rural areas are being surrounded and swallowed by the city. It accelerates the migration between urban and rural areas. Therefore, high construction land area proportion relates to higher transmission and incidence of COVID-19. Besides, Wuhan has always been one of the most important industrial cities in China since 1949. Therefore, there are a large number of old residential buildings in Wuhan. In the rapid urbanization process, a large number of new residential buildings are developed in Wuhan in recent years. Generally speaking, the floor areas of new residential buildings are larger than the floor areas of old residential buildings. The living environment of residential quarters in old residential buildings is crowded. These residential quarters cannot strictly control entrances. According to the previous research of Karako et al. [27], gated community and uncrowded living environment could restrict the transmission of COVID-19. Consequently, the high average building scale can reduce the COVID-19 morbidity rate.

Economic activities have tight impacts on the COVID-19 morbidity rate. GDP per unit of the land area indicates the developing level of the regional economy. The governments and individuals in the areas that have high GDP per unit of land area have more resources to control the transmission of COVID-19. The development of the tertiary industry and retail sales needs a large number of proprietors and consumers. Areas with a high value-added tertiary industry per unit of land area imply that there is an intensive tertiary industry in these areas, and the markets in these areas are flourishing. Areas with total retail sales of consumer goods per unit of the land area indicate that people prefer to purchase in the market in these areas. These influencing factors lead to the risk of the transmission of COVID-19 in the commercial activity space. Therefore, the high value-added tertiary industry per unit of land area and total retail sales of consumer goods per unit of the land area increase the COVID-19 morbidity rate.

Public service status also relates to the COVID-19 morbidity rate. Public green space includes parks, small gardens, squares, botanical gardens, zoos. Areas with high public green space density provide more public green space to people for outdoor activities. In the early stage of the COVID-19 pandemic in Wuhan, people were allowed to do some outdoor activities in the public green space. Consequently, high public green space density increases outdoor activity opportunities, resulting in the increasing opportunities for close contact in public green space. COVID-19 is believed to be primarily spread during close contact [28]. Therefore, high public green space density increases the COVID-19 morbidity rate. COVID-19 has resulted in a horrifying situation in Wuhan in January and February 2020. So, the hospitals in Wuhan have suffered huge pressures in the outbreak of COVID-19 since a large number of patients demanded medical examination and treatment. Hospitals have been important places for efficient person-to-person transmission in this COVID-19 pandemic in Wuhan [15]. Areas with high hospital density denote that there are more hospitals in the unit area, and patients can be scattered among these hospitals. It reduces the risk of person-to-person transmission in hospitals. Consequently, hospital density significantly negatively correlates with the COVID-19 morbidity rate. The elderly population is susceptible to COVID-19 since the underlying disease of the elderly population [29]. Therefore, aged population density can increase the COVID-19 morbidity rate.

### 4.2. Implications for Urban Development

Significant relationships between social and economic factors and the COVID-19 morbidity rate were observed in this study. Notably, it provided some important information to control the transmission of COVID-19 [30]. It benefits the urban development to establish healthy cities in China.

The built environment can be designed to control the transmission of COVID-19 based on its effects on the COVID-19 morbidity rate. We should adopt sustainable land intensive use since high land-use intensity raises the transmission risk of COVID-19. Sustainable land intensive use doesn’t infinitely increase land-use intensity. Therefore, the population per unit area should be reduced in urban centers. Meanwhile, urban planning should reserve agricultural land in cities. We should control the conversion from agricultural land to construction land in the urbanization process [31]. Independent rural areas should be reserved to maintain continuous production and living. Besides, the governments should establish the closed perimeter of walls to improve the closed-off management of old residential quarters in the reconstruction process of old residential quarters. Public space of old residential buildings should be enlarged to alleviate the overcrowding.

Economic activities can be guided to control the transmission of COVID-19 based on their effects on the COVID-19 morbidity rate. Urban development should accelerate economic development. The developed economy is very important to control the COVID-19 pandemic. Due to the impact of e-commerce, land-use forms, including street shops, community retail stores, and small supermarkets, are failed in urban areas. However, these land-use forms are convenient to satisfy the survival needs of urban residents. In order to improve the stability of urban operation during the COVID-19 pandemic, small commercial facilities should be scientifically arranged around residential areas.

Public service status can be optimized to control the transmission of COVID-19 based on its effects on the COVID-19 morbidity rate. Increasing hospitals and other public service facilities is beneficial to control the COVID-19 pandemic. Therefore, it should be combined with transportation, population, natural conditions, and other influencing factors to arrange public service facilities. But public service facilities, such as public green space and nursing homes, are short of the measures to prevent the spread of disease. Public service facilities also accelerate the transmission of the pandemic. Therefore, we should keep sufficient spatial redundancy to solve densely populated public service facilities based on population flow prediction. Besides, we should arrange sufficient sanitary facilities in the public service facilities.

### 4.3. Method Limitations

However, we must acknowledge the limitations of our work. First, on 17 April 2020, the Chinese government stated that the number of deaths from COVID-19 was 3869 instead of 2579. We believe that some confirmed cases of COVID-19 were missed on 22 February 2020. However, we also believe that the Chinese government reported the vast majority of confirmed cases of COVID-19 in Wuhan on 22 February 2020. Besides, asymptomatic cases of COVID-19 were not considered in the study since the Chinese government did not report the number of asymptomatic cases of COVID-19 on 22 February 2020. Up to now, nobody has known the accurate data of asymptomatic cases of COVID-19 in Wuhan. Second, some data on social and economic factors were surveyed before the outbreak of COVID-19 in Wuhan. It was hard to survey the data in Wuhan during the outbreak of COVID-19. Third, heterogeneity among different districts in Wuhan was not considered in this study. It might result in the deviation of the relationships between social and economic factors and COVID-19 spread. Forth, there were logistical difficulties in pinpointing the mechanism between social and economic factors and COVID-19 spread. We didn’t assess the mediating effects between social and economic factors and the COVID-19 morbidity rate. Future studies should adopt other research methods and combine mediating variables to deepen the knowledge between social and economic factors and COVID-19. Moreover, we need to survey the latest COVID-19 morbidity rate and precise data on social and economic factors during the ongoing outbreak of COVID-19.

## 5. Conclusions

Using the case of Wuhan, this study analyzed the distribution of the COVID-19 morbidity rate in association with social and economic factors. Specifically, social and economic factors were classified into three dimensions: built environment, economic activities, and public service status. The method applied was spatial regression analysis. Innovative contributions had been achieved in two aspects: (1) social and economic factors that have impacts on COVID-19 morbidity rate were surveyed in Wuhan, and (2) the distribution of COVID-19 morbidity rate in association with social and economic factors was quantified. These findings could offer practical insights for urban development.

## Figures and Tables

**Table 1:** COVID-19 morbidity rate in Wuhan.

District	Confirmed Case (Person)	Average Population (10^4^ Person)	COVID-19 Morbidity Rate (‱)
Jiang’an District	4117	85.21	48.32
Jianghan District	7099	61.29	115.82
Qiaokou District	6500	69.86	93.05
Hanyang District	3172	55.68	56.96
Wuchang District	7873	116.02	67.86
Qingshan District	2815	47.75	58.95
Hongshan District	4648	135.55	34.29
Dongxihu District	2523	43.77	57.65
Wuhan development zone including Hannan District	1470	43.44	33.84
Caidian District	1854	37.39	49.58
Jiangxia District	1465	76.26	19.21
Huangpi District	1669	106.08	15.73
Xinzhou District	996	93.17	10.69

**Table 2:** Variable selection.

Dimension	Variables	Definitions	Units
	COVID-19 morbidity rate(COV)	The ratio of confirmed cases of COVID-19 to the average population	‱
Built environment	Population density(POD)	The ratio of the resident population to construction land area	10^4^ persons/km^2^
	Construction land area proportion(CLP)	The ratio of construction land area to land area	-
	Average building scale(ABS)	The ratio of total floor area to the number of residential buildings	m^2^
Economic activities	GDP per unit of land area(GPA)	Ratio between gross domestic product and land area	10^9^ Yuan/km^2^
	Value-added of tertiary industry per unit of land area(VTA)	Ratio between value-added of tertiary industry and land area	10^9^ Yuan/km^2^
	Total retail sales of consumer goods per unit of land area(TRA)	Ratio between total retail sales of consumer goods and land area	10^9^ Yuan/km^2^
Public service status	Public green space density(PGD)	Per capita public green space	m^2^/person
	Hospital density(HOD)	The ratio of number of hospitals to land area	person/km^2^
	Aged population density(APD)	The ratio of population aged 65 and over to the land area	person/km^2^

**Table 3:** Descriptive statistics of variables (*N* = 13). COV (COVID-19 morbidity rate), POD (Population density), CLP (Construction land area proportion), ABS (Average building scale), GPA (GDP per unit of land area), VTA (Value-added of tertiary industry per unit of land area), TRA (Total retail sales of consumer goods per unit of land area), PGD (Public green space density), HOD (Hospital density), APD (Aged population density).

Variables	Units	Min.	Max.	Mean	Std.dev.
COV	‱	10.691	115.818	50.919	30.082
POD	10^4^ persons / km^2^	0.214	2.693	1.175	0.949
CLP	-	0.085	0.958	0.463	0.310
ABS	m^2^	300.770	2380.376	1056.764	635.123
GPA	10^9^ Yuan / km^2^	0.311	40.388	8.680	11.439
VTA	10^9^ Yuan / km^2^	0.094	37.744	6.560	10.737
TRA	10^9^ Yuan / km^2^	0.120	38.876	7.067	11.751
PGD	m^2^ / person	3.600	15.550	10.280	3.396
HOD	person / km^2^	0.007	1.198	0.330	0.452
APD	person / km^2^	27.242	3026.261	794.208	991.158

**Table 4:** Regression table of the distribution of COVID-19 morbidity rate in association with social and economic factors. COV (COVID-19 morbidity rate, ‱), POD (Population density, 10^4^ persons/km^2^), CLP (Construction land area proportion, -), ABS (Average building scale, m^2^), GPA (GDP per unit of land area, 10^9^ Yuan/km^2^), VTA (Value-added of tertiary industry per unit of land area, 10^9^ Yuan/km^2^), TRA (Total retail sales of consumer goods per unit of land area, 10^9^ Yuan/km^2^), PGD (Public green space density, m^2^/person), HOD (Hospital density, person/km^2^), APD (Aged population density, person/km^2^).

	Dependent variable: COV		
	OLS	SLM	SEM
CONSTANT	0.229	−38.862 **	29.551
	(0.996)	(0)	(0.014)
POD	5.279	38.338 **	−148.779
	(0.943)	(0.008)	(0)
CLP	63.849	57.859 **	58.292
	(0.583)	(0.009)	(0.345)
ABS	−0.019	−0.025 **	−0.008
	(0.641)	(0.001)	(0.442)
GPA	1.489	−4.586 *	4.482
	(0.875)	(0.013)	(0.395)
VTA	−1.474	4.360 **	5.045
	(0.860)	(0.008)	(0.403)
TRA	3.130	4.479 **	−6.939 **
	(0.685)	(0.003)	(0)
PGD	2.397	2.079 **	5.148 **
	(0.455)	(0)	(0.001)
HOD	−39.743	−186.680 **	208.303 **
	(0.851)	(0)	(0)
APD	−0.002	0.021 *	0.045 **
	(0.959)	(0.021)	(0.003)
*λ*/*ρ*		0.934 **	−1.605 **
		(0)	(0)
*R* ^2^	0.880	0.977	0.982
Log likelihood	−48.417	−40.500	−46.572
Akaike info criterion	116.834	103.000	113.145
Lagrange Multiplier (lag)	5.447 **		
	(0.020)		
Robust LM (lag)	10.858 **		
	(0.001)		
Lagrange Multiplier (error)	0.033		
	(0.857)		
Robust LM (error)	5.444 *		
	(0.020)		

* *p* < 0.05, ** *p* < 0.01. OLS (least square estimation), SLM (spatial lag model), SEM (spatial error model).

## Supplementary Materials

The following are available online at https://www.mdpi.com/1660-4601/17/10/3417/s1, the supplementary materials include the data sources, the units of analysis, and the selected software.
