# Supplementary material for: Distribution of COVID-19 Morbidity Rate in Association with Social and Economic Factors in Wuhan, China: Implications for Urban Development
Source: Int J Environ Res Public Health. 2020 May 14;17(10):3417. doi: 10.3390/ijerph17103417 (PMC7277377; doi:10.3390/ijerph17103417)
Supplement: Supplementary file 1 [file ijerph-17-03417-s001.pdf]

Supplemental material

1. Data sources

Table S1 Data sources

Dimension	Variables	Definitions	Units	Sources
	COVID-19 morbidity rate （COV）	The ratio of confirmed cases of COVID-19 to the average population	‰	WeChat official account of the Wuhan government The deadline date of confirmed cases of COVID-19 is February 22, 2020
Built environment	Population density（POD）	The ratio of the resident population to construction land area	10^4 persons / km^2	Wuhan Statistical Yearbook (2018)
	Construction land area proportion（CLP）	The ratio of construction land area to land area	-	Department of land resources of Hubei province
	Average building scale（ABS）	The ratio of total floor area to the number of residential buildings	m^2	Wuhan Construction Yearbook (2018)
Economic activities	GDP per unit of land area （GPA）	Ratio between gross domestic product and land area	10^9 Yuan / km^2	Wuhan Statistical Yearbook (2018)
	Value-added of tertiary industry per unit of land area （VTA）	Ratio between value-added of tertiary industry and land area	10^9 Yuan / km^2	Wuhan Statistical Yearbook (2018)
	Total retail sales of consumer goods per unit of land area （TRA）	Ratio between total retail sales of consumer goods and land area	10^9 Yuan / km^2	Wuhan Statistical Yearbook (2018)
Public service status	Public green space density （PGD）	Per capita public green space	m^2 / person	Wuhan Construction Yearbook (2017)
	Hospital density（HOD）	The ratio of number of hospitals to land area	person / km^2	Wuhan Health Yearbook (2018)
	Aged population density （APD）	The ratio of population aged 65 and over to the land area	person / km^2	Wuhan Health Yearbook (2018)

2. Units of analysis

Administrative divisions that refer to 13 districts in Wuhan are applied in the spatial regression analysis in this study.

Table S2 Units of analysis

**District**	**Confirmed case (person)**	**Average population (10^4 person)**	**Area (km2)**
Jiang'an District	4117	85.21	64.24
Jianghan District	7099	61.29	33.43
Qiaokou District	6500	69.86	46.39
Hanyang District	3172	55.68	108.34
Wuchang District	7873	116.02	87.42
Qingshan District	2815	47.75	68.40
Hongshan District	4648	135.55	480.20
Dongxihu District	2523	43.77	439.19
Wuhan development zone including Hannan District	1470	43.44	489.70
Caidian District	1854	37.39	906.10
Jiangxia District	1465	76.26	2010.00
Huangpi District	1669	106.08	2261.00
Xinzhou District	996	93.17	1500.00

3. Software

Pearson correlation analysis was performed in SPSS. And the graphs of Pearson correlation analysis were mapped in SigmaPlot.

Geoda was selected to analyze spatial regression in this study. The spatial correlation test for the COVID-19 morbidity rate was also performed in Geoda.
